# An Investigation on the Microstructure and Texture of an AZ80 Cup-Shaped Piece Processed by Rotating Backward Extrusion

**DOI:** 10.3390/ma13173690

**Published:** 2020-08-20

**Authors:** Xin Che, Beibei Dong, Kai Liu, Qiang Wang, Mu Meng, Zhi Gao, Jin Ma, Fulai Yang, Zhimin Zhang

**Affiliations:** College of Materials Science and Engineering, North University of China, Taiyuan 030051, China; CXIN19931029@163.com (X.C.); dongbb1111@163.com (B.D.); LK752611164@163.com (K.L.); mengmu@nuc.edu.cn (M.M.); SZHENGNUC@163.com (Z.G.); nucmajin@163.com (J.M.); nucyangfulai@163.com (F.Y.)

**Keywords:** AZ80 magnesium alloy, rotating backward extrusion, severe plastic deformation, microstructure, texture

## Abstract

The rotating backward extrusion (RBE) process, as a new severe plastic deformation (SPD) process, is based on conventional backward extrusion and rotation, which meets the requirement of modern industrial development with its high performance and production efficiency. However, there is little research on the microstructure evolution and texture modification of the RBE process. Thus, in this study, the effect of different rotating revolutions, e.g., *n* = 5, *n* = 10, and *n* = 50, on the microstructure and texture development for the RBE process based on the AZ80 magnesium (Mg) alloy were investigated at 653 K. The results disclose that the rotating revolution is an influencing processing parameter on the deformation of the RBE process. The grain refining ability is enhanced with the increase of the rotating revolutions, and the minimum grain size of the cup bottom, shearing zone, and cup wall can reach to 16.7 μm, 15.6 μm, and 13.0 μm, respectively, under the condition of *n* = 50. Furthermore, with the increase in the rotating revolutions, the microstructure of the alloy becomes more uniform and the proportion of dynamic recrystallization (DRX) is also increased. The maximum DRX fractions of the sample for the cup bottom and cup wall are 95.4% and 86.8%, respectively, at *n* = 50. The DRX mechanism of the RBE process is determined by the continuous DRX and discontinuous DRX. In addition, the texture can be significant weakened during the RBE process, especially at the cup bottom, where the maximum pole intensity can be reduced from 17.6 at *n* = 10 to 6.5 at *n* = 50, which can be attributed to the higher proportion of new DRXed grains whose orientations are more random compared with the deformed grains.

## 1. Introduction

Magnesium (Mg) alloys have an important application prospect in many fields, owing to their low density, high specific strength, and good damping [1,2,3,4]. However, Mg alloys usually exhibit a poor ductility and forming ability at an ambient temperature because of the special hexagonal close-packed (HCP) structure [5,6,7]. Lei et al. [4] reported that grain refinement and texture controlling were two effective ways to enhance the strength of Mg alloys. Therefore, in order to obtain high-performance Mg alloys, it is urgent to develop some new forming processes to refine grains and weaken texture.

As we all know, the current severe plastic deformation (SPD) method is an efficacious way to refine grains and weaken texture during the thermal process. SPD methods mainly include high pressure torsion (HPT) [8], multi-directional forging (MDF) [9], accumulative roll-bonding (ARB) [10], accumulative back extrusion (ABE) [11], friction stir processing (FSP) [12], equal channel angular processing (ECAP) [13], differential speed rolling (DSR) [14], repetitive upsetting extrusion (RUE) [15], and so on. All of the above SPD methods can refine grains, weaken texture, and enhance the mechanical properties of the materials significantly. However, the above experimental methods need to increase the cumulative strain through multi-pass deformation during the thermal deformation in order to achieve the purpose of refining grains, weakening the texture, and improving mechanical performance. Therefore, in order to save production costs, improve production efficiency, and overcome the deficiencies in the above SPD methods, the combination process that introduces torsion into conventional extrusion (CE) is an effective method to solve this problem. Mohammad et al. [16] applied torsion to the forward extrusion via die rotating, and this method could produce a strong continuous shear strain on the extruded material during one cycle. Kong et al. [17] found that the tensile toughness of the experimental alloy was enhanced via torsion extrusion at room temperature compared with the other deformation methods. Korbel et al. [18] developed a new RE process named the “KOBO” method, by changing the deformation path, i.e., die rotated from one-way rotation to reciprocating cycle rotation, and found that the grain refining ability and mechanical performance were both enhanced during the KOBO process.

**Figure 1:**
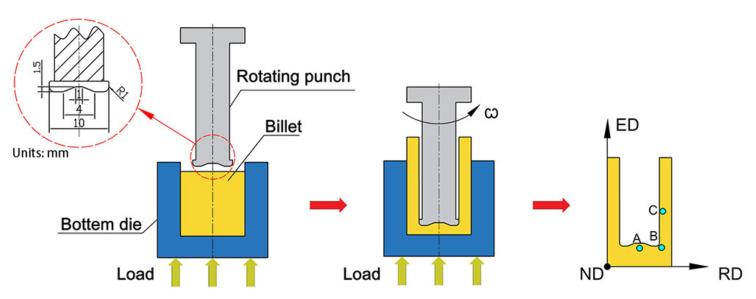
Schematic illustration and microstructure observation position of the rotating backward extrusion (RBE) process.

In recent years, we have developed a new SPD technique titled rotating backward extrusion (RBE), which can produce ultra-fine grain (UFG) parts [1,19,20]. The RBE process is characterized by the rotation of an open punch during the thermal extrusion process, which can reduce the extrusion load and produce a large accumulative plastic strain through single pass deformation. The schematic illustration of the RBE process is shown in Figure 1. It can be found that the RBE method consists of an open punch with a transverse groove at the end face of the punch, which can lead more materials to be rotated.

Thus, it can better illuminate the deformation behavior during the RBE process rather than being limited to the forming ability of the RBE technology. In this paper, both the microstructure and texture evolution during the RBE process are investigated, and the deformation parameters mainly include the deformation temperature of 653 K and the rotating revolutions of *n* = 5, *n* = 10, and *n* = 50, respectively. According to the finite element simulation results from our previous work [20], the inner wall of the cup is the area where the equivalent strain changes the most. Furthermore, considering the characteristics of backward extrusion deformation, the metal starts from the bottom of the sample, flows through the shear zone, and finally forms the wall. Therefore, selecting these three regions can accurately characterize the metal flow of the RBE process.

## 2. Materials and Methods

The experimental alloy was an as-cast commercial AZ80 Mg alloy provided by Wenxi Yinguang of Magnesium Industry Co., Ltd. (Wenxi, Shanxi, China), made via the semi-continuous casting process, and the chemical composition of the studied alloy is exhibited in Table 1. Then, the as-cast ingot was homogenized at a temperature of 688 K with a holding time of 16 h. The size of the cylindrical specimen was Φ 25 mm × 22 mm, taken out from the as-cast ingot. Finally, the homogenized alloy was quenched in water. The oil-based graphite was chosen as the lubricant in order to reduce the friction between the dies and the billet. The RBE experiment was conducted at 653 K, with a die speed of 0.05 mm/s, using the Gleeble-3500 thermal simulation test machine developed by DSI Company (New York, NY, USA) and the Precision Forming Center of North University of China. The die first moved 2 mm to make the material flow into the transverse groove of the open punch. Then, the open punch continued to move and rotated at the same time until the end of the deformation, and finally the cup-shaped specimen was obtained. When the whole RBE process was finished, the RBE specimens were immediately quenched in air to retain the high temperature deformation microstructure. In our previous research [1], it could be found that the rotating speeds of the RBE specimens were 0.079, 0.785, and 1.57 rad/s, respectively, which corresponded to rotating revolutions of *n* = 5, *n* = 10, and *n* = 50.

The observation planes were chosen in a direction parallel to the extrusion direction (ED) of the RBE specimens, with rotating revolutions of *n* = 5, *n* = 10, and *n* = 50, respectively. Regions A, B, and C were selected to study the microstructure and texture evolution after the RBE process, as shown in Figure 1. The three regions mentioned above were carried out from the final cup-shaped specimen, with a size of 5 × 5 × 5 mm^3^. Once the observation samples were machined from the cup-shaped specimens after RBE process, the samples were ground using 600, 1500, 3000, 5000, and 7000 SiC papers, and mechanically polished with Al_2_O_3_ suspension solutions, followed by etching with a chemical reagent of 0.3 g oxalic acid, 3 ml acetic acid, and 20 ml distilled water. The microstructure and texture were observed using an optical microscope (OM; Zeiss Axio Imager-A2m, Oberkochen, Germany) and scanning electron microscopy (SEM; Hitachi-SU5000, Tokyo, Japan) equipped with electron backscatter diffraction (EBSD), supplied by the EDAX company (EDAX Inc., Mahwah, NJ, USA). The samples were pre-polished on a precision grinding machine (Leica EM-TXP, Wetzlar, Germany) and polished on an argon ion polishing machine (Leica EMS-102, Wetzlar, Germany) with a voltage of 6.5 kV and electric current of 2.5 mA before EBSD analysis. The parameters for the EBSD observation were a working distance of 15 mm, tilt of 70°, and step size of 0.15–1.0 μm. The EBSD data were analyzed by orientation imaging microscopy (OIM) software 7 (EDAX Inc., Mahwah, NJ, USA). Moreover, the grain size and the area fraction of the dynamic recrystallized (DRX) grains was measured using the EBSD data (EDAX Inc., Mahwah, NJ, USA).

## 3. Results and Discussion

### 3.1. Microstructure Evolution

**Figure 2:**
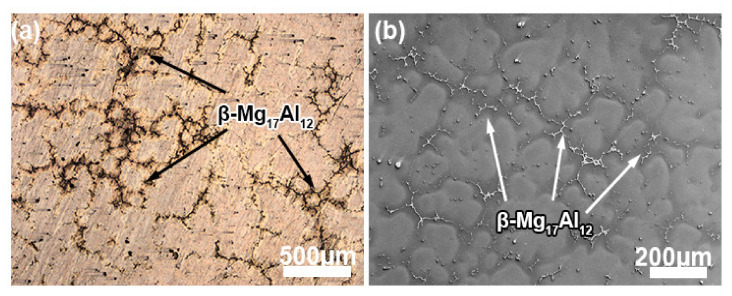
The (**a**) OM and (**b**) SEM images of the as-homogenized AZ80 alloy.

Figure 2 shows the optical microscopes (OM) and SEM images of the as-homogenized alloy. It can be observed that the coarse initial grains, with a grain size of nearly 300 μm, are surrounded by the undissolved eutectic phase, which has been confirmed to be the β-Mg_17_Al_12_ phase in our previous work [20]. It is indicated that the Al atoms inside the grains diffuse more easily than those at the grain boundaries during the homogenization process.

**Figure 3:**
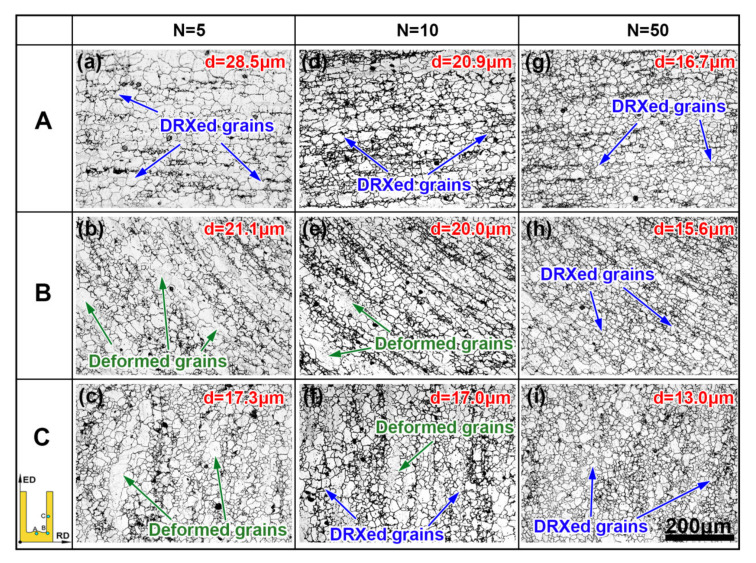
The OM maps of the RBE samples at the three different locations: (**a**–**c**) *n* = 5, (**d**–**f**) *n* = 10, and (**g**–**i**) *n* = 50.

Figure 3 displays the microstructure of the RBE samples from the cup bottom to the cup wall with different rotating revolutions. It can be observed from Figure 3 that the microstructure gradually changed with the material flow toward the die exit during deformation, in the same rotating revolution. Some initial grains are elongated along the metal flow direction, but the other coarse initial grains are completely engulfed by the new fined DRXed grains. The average grain size for all of the samples is smaller than 30 μm. For the same revolutions, the microstructure uniformity and grain refinement are improved from regions A to C, and the largest reduction in grain size is 39.3% for the *n* = 5 sample. The same phenomena can also be obtained in the condition for the same region, and the grain size reductions for regions A to C are 41.4%, 26.0%, and 24.9%, respectively, from *n* = 5 to *n* = 50. It is noted that the results of the grain refinement from regions A to C are related to the shear strain caused by the metal reverse flow. On other hand, the grain size decreases with the rotating revolutions increasing in the same region, which can be attributed to the additional torsion during the RBE process. The simulation results in our previous work [20] suggested that increasing the rotating revolutions can significantly improve the average effective strain of the alloys. The large accumulative strain can not only promote the formation and movement of the dislocations, but also contribute to more non-basal slip systems being activated [21]. The pile ups of dislocations can provide more nucleation sites for the DRXed grains, and the cross-slip of dislocation between the basal plane and the non-basal plane slip systems can lead to the formation of low angle grain boundaries (LAGBs), which ultimately contributes to the occurrence of DRXed grains [22,23]. Because the sample in region A exhibits the largest grain size of 28.5 μm at *n* = 5, the change of grain size is the most significant in this region. Therefore, it can be inferred that the grain refining ability can be significantly enhanced during the RBE process.

**Figure 4:**
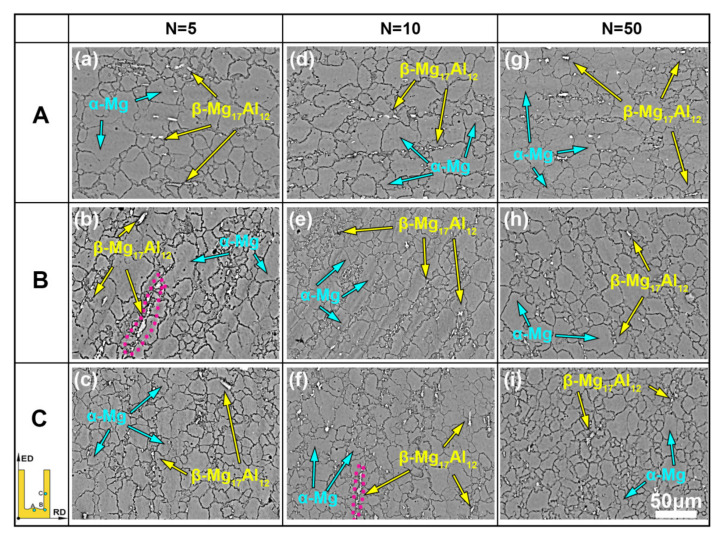
The BSE images of the RBE samples at the three different locations: (**a**–**c**) *n* = 5, (**d**–**f**) *n* = 10, and (**g**–**i**) *n* = 50.

Figure 4 presents the backscatter electron (BSE) images of the RBE samples at different regions. It is well known that the different color contrast in the BSE maps represent different phases. There are two different contrasts—the dark contrast of the Mg matrix and the bright contrast of the second phase. The XRD results in our previous studies [20] have demonstrated that these second phases belong to the β-Mg_17_Al_12_ phase, that is, the type of the second phase will be not changed with the number of revolutions increasing. It can be seen that the continuous β phases in the initial grain boundaries, with a length of about 100 μm, are broken into a smaller phase with a size of less than 10 μm during RBE deformation. Moreover, no dynamic precipitation phases with a size of 1μm can be observed because of the high solid solubility of Al atoms at 653 K, which is not conducive to precipitation. In addition, there are still some large-sized β phases in the low revolution samples (highlighted by red dotted circles in Figure 4b,f), but as the number of rotating revolutions increases, these remaining coarse phases also gradually break into small-sized phases (Figure 4g–i). In addition, it should be noted that the β phases are mostly distributed along the DRXed grain boundaries rather than inside the grain, which can effectively inhibit the grain growth, that is, creating the pinning effect [24]. Therefore, in the RBE deformation, increasing the number of revolutions can reduce the size of the second phase, thereby reducing the size of the DRXed grains, and ultimately achieving the effect of grain refinement.

**Figure 5:**
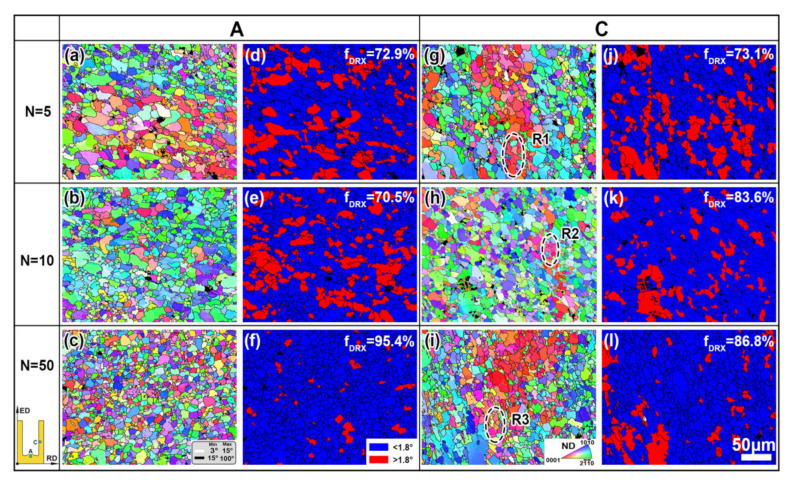
The inverse pole figure (IPF) and corresponding grain orientation spread (GOS) maps of the RBE samples at the two different locations: (**a**,**d**,**g**,**j**) *n* = 5, (**b**,**e**,**h**,**k**) *n* = 10, and (**c**,**f**,**i**,**l**) *n* = 50. (For interpretation of the references to color in this figure legend, the reader is referred to the Web version of this article.)

Figure 5 shows the EBSD results of the RBE samples in regions A and C with different rotating revolutions. The black regions in the inverse pole figure (IPF) maps and the grain orientation spread (GOS) maps represent the β phases, which cannot be defined because of the lack of phase data. Meanwhile, the LAGBs (misorientation angles between 3° and 15°) are marked with white lines and the high angle grain boundaries (HAGBs; misorientation angles higher than 15°) are marked with black lines. In this study, GOS was selected to analyze the DRX fraction—the critical GOS value for DRX grains is identified as 1.8° [25], as shown in Figure 5d–f,j–l.

It can be seen from the IPF maps (Figure 5a–c,g–i) that the grain size is decreased and the microstructure uniformity is enhanced with the revolutions increasing in the same region, which is the same as for the microstructure change trend in the OM and BSE observations. Furthermore, lots of LAGBs can be obtained in both deformed and DRXed grains, indicating a high activity of dislocations. The formation of LAGBs is related to the local lattice rotation caused by the uneven local deformation inner grains, and the orientation difference can easily cause pile ups of dislocation, which in turn lead to a local stress concentration. With the progress of deformation, the orientation difference in the grain interior gradually is increased to LAGBs, and finally to HAGBs [22]. Thus, the large strain implied by the additional rotation can promote the formation of dislocations, which can increase the amount of LAGBs and promote the occurrence of DRXed grains. This conclusion can also be demonstrated by the changing trend of the GOS maps.

It can be seen from the GOS maps (Figure 5d–f,j–l) that the DRX fraction increase in revolutions in each region, and it also increases from regions A to C in the same revolution, except for the condition of *n* = 50. The largest DRX fraction for regions A and C is 95.4% and 86.8%, respectively, at *n* = 50. It is noted that the DRX fraction is larger than 70% for all of the samples; this is because the higher temperature provides a sufficient driving force for the growth of DRX. Increasing the rotating revolutions, however, can increase the dislocation activity of the inner grain, leading to more nucleation site for the occurrence of DRXed grains, thus the proportion of the DRXed grains is significantly increased. For the *n* = 50 sample, a large amount of frictional heat may be generated because of the large rotating speed, which promotes the abnormal growth of deformed grains, resulting in a decrease in the area fraction of DRXed grains. Therefore, for the RBE process, increasing the rotation speed can promote grain refinement and improve the proportion of DRXed grains, but excess friction heat will also affect the grain refinement effect, so how to control the transfer of friction heat is also an important problem to be solved for the RBE process, which needs more attention in future research.

**Figure 6:**
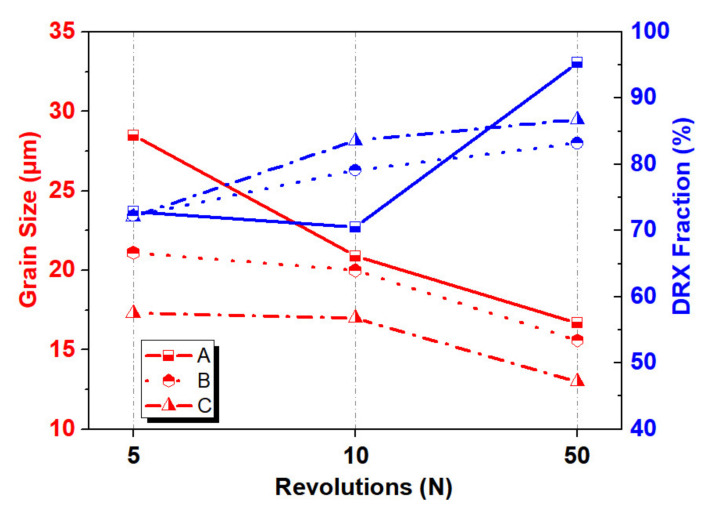
The comparison of average grain size and DRX fraction with different rotating revolutions and locations.

Figure 6 shows a comparison of the average grain size and DRX fraction at different rotating revolutions and locations. It can be seen that the change trend of the average grain size is opposite to the change trend of the DRX fraction, in which the maximum grain size is reduced from 300 μm for the initial grains, to 13 μm for the *n* = 50 sample with a decrease of about 95.7%. Therefore, it can be concluded that increasing the number of rotating revolutions is an effective way to promote grain refinement and microstructure uniformity.

### 3.2. DRX Behaviors during the RBE Process

**Figure 7:**
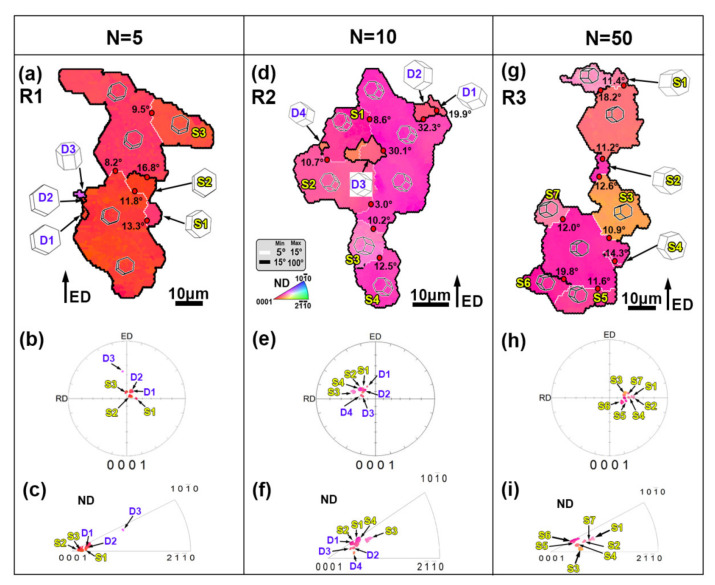
The DRX mechanism of the RBE samples in the unDRX zones (**a**–**c**) R1, (**d**–**f**) R2, and (**g**–**i**) R3 selected in Figure 5g–i: (**a**,**d**,**g**) inverse pole figure (IPF) maps, the corresponding orientation highlighted in (**b**,**e**,**h**) (0001) pole figure (PF), and (**c**,**f**,**i**) inverse pole figure (IPF). S-subgrain; D-DRXed grain.

In order to illustrate the deformation mechanism of the AZ80 alloy during the RBE process, three unDRXed zones are selected from region C, based on the EBSD data (R1–3 in Figure 5g–i). Figure 7 shows the DRX behaviors of the RBE samples at different rotating revolutions. The IPF map of R1 (Figure 5a) shows that the initial grain boundaries (GBs) bulge outward, leaving a LAGB inner grain, such as S1–S3, which is a typical feature of the discontinuous DRX (DDRX). It is well known that DDRX is related to strain-induced grain boundary migration, which leads the grain nucleation and growth through the serrate GBs [26,27]. Hadorn et al. [28,29] report that DDRXed grains inherit the grain orientation from their parent grains. However, it can be obtained from the corresponding (0001) PF and IPF of R1 (Figure 7b,c) that DDRXed grains such as D1 and D2 exhibit the same orientation as their parent grains, but D3 has a rotated orientation from the parent grains, which can make the texture of the alloys more random.

For region R2 (Figure 7d), some DRXed grains, such as D1, D2, and D4, are also formed based on the serrated GBs, which belong to the DDRXed grains. In addition to these grains, a new DRXed grain (D3) surrounded by LAGBs is formed in the inner grain, which is related to the continuous DRX (CDRX). The CDRX continues through a recovery process with dislocation rearrangement to form subgrains, and the continuous absorption of dislocations in LAGBs finally leads to the formation of HAGBs and new DRXed grains [30,31]. Furthermore, the grain orientation of DRXed grains in region R2 is similar to the subgrains (Figure 7e,f). Combined with the DRXed grain orientation in region R1, it can be inferred that some DRXed grains with the same orientation as the parent grains will be rotated during further deformation, which can ultimately weaken the texture of the alloys. As can be observed from region R3 (Figure 7g), some subgrains (S1, S3, and S5–S7) are formed with the bulge of the GBs, and some subgrains (S2, S4) are formed with LAGBs in the inner grain, indicating the CDRX and DDRX both occur in region C at *n* = 50. Furthermore, the subgrains always have a similar orientation to their parent grains. Therefore, the microstructure evolution of the RBE process is mainly dominated by the deformation mechanism of CDRX and DDRX.

### 3.3. Texture Development

**Figure 8:**
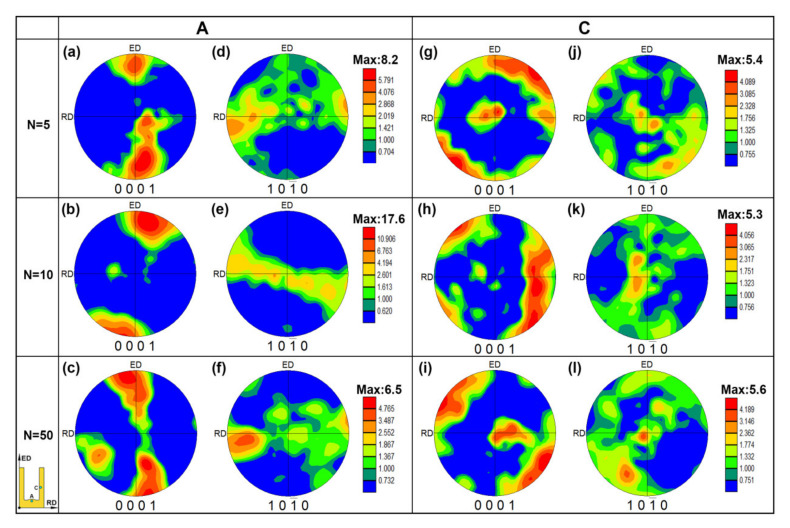
Texture development during the RBE process: (**a**–**c**,**g**–**i**) (0001) pole figures (PF) and (**d**–**f**,**j**–**l**) (10-10) pole figures (PF).

The texture development of the RBE sample in regions A and C at the different rotating revolutions is presented in Figure 8. Figure 8a–f shows that the grain orientations of most of the grains in region A are distributed in an ED and normal direction (ND) plane, which is a typical texture type of the high pressure torsion [32]. Because the basal plane slip is the primary slip system during deformation, and the basal plane of the grain will rotate perpendicular to the direction of the external force (i.e., ED). Furthermore, it should be noted that the maximum pole intensity (MPI) is increased from 8.2 for *n* = 5 to 17.6 for *n* = 10, and then decreased to 6.5 for *n* = 50, which indicates that increasing the revolutions can promote the texture weakening. This phenomena may be attributed to the new DRXed grains that have a more random orientation compared with their parent grains [15]. Moreover, the proportion of DRXed grains in region A shows a trend of decreasing first and then increasing with the increase of rotating revolutions, which is exactly the opposite to the change trend of MPI. Therefore, it can be concluded that increasing the rotating revolutions during the RBE process can promote the ability of grain refinement and texture weakening. In addition, it can be observed from region C (Figure 8g–l) that the basal planes of most of the grains are rotated from the ED to radial direction (RD), which is caused by the metal reverse flow. The MPI of region C is smaller than that of region A in the same revolution, but the MPI is not changed significantly in region C with different revolutions. Although increasing the rotation speed will increase the DRX fraction in region C, the samples have a higher DRX fraction in this region and most of the new DRXed grains may be rotated to a direction perpendicular to the external force during the reverse metal flow, thus there will be no significant texture weakening in region C.

## 4. Outlook

For the RBE process, there are two aspects that will be investigated in the future. One is to further reduce the relative sliding between the mold and the billet, so that more torque can be transformed to the billet, bringing more deformations to the billet. For the first aspect, developing a new open punch with different amounts of grooves may solve this problem. In the other aspect, it is necessary to apply the deformation characteristic obtained in the research of small-size specimens to the development of large-size components in order to promote the industrial application of the RBE process.

## 5. Conclusions

In the present study, a novel SPD process titled rotating backward extrusion (RBE), with an open punch, is introduced. The effects of the different rotating revolutions on the evolution of the microstructure and texture of the AZ80 alloy cup-shaped piece are investigated. The main conclusions are summarized as follows:

1. The grain refinement and microstructure uniformity can be enhanced by increasing the rotating revolutions. Furthermore, the large β phase can be broken into a small size phase with the increase of revolutions.
2. The DRX fraction of the alloy can be increased with the revolutions increasing, and the main deformation mechanism of the RBE process is the CDRX and DDRX.
3. Increasing the rotating revolutions can effectively weaken the texture during the RBE process, which may be ascribed to the occurrence of new DRXed grains with a random orientation.

## Figures and Tables

**Table 1:** Chemical compositions of the experimental AZ80 alloy.

Al	Zn	Mn	Cu	Fe	Ni	Si	Mg
8.43	0.60	0.19	<0.05	<0.05	<0.05	<0.05	Bal.

## References

[1] Dong B.B., Che X., Wang Q., Meng M., Gao Z.J., Ma F.L., Yang Z., Zhang M. (2020). Refining the microstructure and modifying the texture of the AZ80 alloy cylindrical tube by the rotating backward extrusion with different rotating revolutions. J. Alloys Compd..

[2] You S.H., Huang Y.D., Kainer K.U., Hort N. (2017). Recent research and developments on wrought magnesium alloys. J. Magnes. Alloy.

[3] Victoria-Hernandez J., Yi S., Letzig D., Hernandez-Silva D., Bohlen J. (2013). Microstructure and texture development in hydrostatically extruded Mg-Al-Zn alloys during tensile testing at intermediate temperatures. Acta Mater..

[4] Lei J.Y., Ma L.F., Jia W.T., Zhu Y.C., Huang Z.Q., Lin J.B. (2019). Microstructure and properties of Mg-Al-Mn magnesium alloy under shear deformation coupled tension rolling. Mater. Res. Express.

[5] Wang C.P., Mei H.S., Li R.Q., Li D.F., Wang L., Hua Z.H., Zhao L.J., Pen F.F., Li H. (2013). Microstructure evolution and grain coarsening behaviour during partial remelting of cyclic extrusion compression formed AZ61 magnesium alloy. Acta Metall. Sin..

[6] Du B.N., Hu Z.Y., Sheng L.Y., Xu D.K., Zheng Y.F., Xi T.F. (2018). Influence of Zn content on microstructure and tensile properties of Mg-Zn-Y-Nd alloy. Acta Metall. Sin..

[7] Cheng W.L., Wang L.F., Zhang H., Cao X.Q. (2018). Enhanced stretch formability of AZ31 magnesium alloy thin sheet by pro-crossed twinning lamellas induced static recrystallization. J. Mater. Process. Technol..

[8] Zhiyaev A.P., Langdon T.G. (2008). Using high-pressure torsion for metal processing: Fundamentals and applications. Prog. Mater. Sci..

[9] Biswas S., Suwas S. (2012). Evolution of sub-micron grain size and weak texture in magnesium alloy Mg-3Al-0.4Mn by a modified multi-axial forging process. Scr. Mater..

[10] Roostaei A.A., Zarei-Hanzaki A., Abedi H.R., Rokni M.R. (2011). An investigation into the mechanical behavior and microstructural evolution of the accumulative roll bonded AZ31 Mg alloy upon annealing. Mater. Des..

[11] Fatemi-Varzaneh S.M., Zarei-Hanzaki A. (2009). Accumulative back extrusion (ABE) processing as a novel bulk deformation method. Mater. Sci. Eng. A.

[12] Razmpoosh M.H., Zarei-Hanzaki A., Imandoust A. (2015). Effect of the Zenere Hollomon parameter on the microstructure evolution of dual phase TWIP steel subjected to friction stir processing. Mater. Sci. Eng. A.

[13] del Valle J.A., Carreno F. (2016). Influence of texture and grain size on work hardening and ductility in magnesium-based alloys processed by ECAP and rolling. Acta Mater..

[14] Kim W.J., Park J.D., Kim W.Y. (2008). Effect of differential speed rolling on microstructure and mechanical properties of an AZ91 magnesium alloy. J. Magnes. Alloy.

[15] Zhang G.S., Zhang Z.M., Li X.B., Yan Z.M., Che X., Yu J.M., Meng Y.Z. (2019). Effects of repetitive upsetting-extrusion parameters on microstructure and texture evolution of Mg-Gd-Y-Zn-Zr alloy. J. Alloys Compd..

[16] Mizunuma S. (2006). Large straining behavior and microstructure refinement of several metals by torsion extrusion process. Mater. Sci. Forum.

[17] Kong L.X., Lin L., Hodgson P.D. (2001). Material properties under drawing and extrusion with cyclic torsion. Mater. Sci. Eng. A.

[18] Korbel A., Pospiech J., Bochniak W., Tarasek A., Ostachowski P., Bonarski J. (2011). New structural and mechanical features of hexagonal materials after room temperature extrusion using the KoBo method. Int. J. Mater. Res..

[19] Yu J., Zhang Z., Wang Q., Hao H., Cui J., Li L. (2018). Rotary extrusion as a novel severe plastic deformation method for cyclindrical tubes. Mater. Lett..

[20] Che X., Wang Q., Dong B.B., Meng M., Zhang Z.M. (2020). Numerical and experimental analysis of rotating backward extrusion as a new SPD process. Met. Mater. Int..

[21] Chapuis A., Driver J.H. (2011). Temperature dependency of slip and twinning in plane strain compressed magnesium single crystals. Acta Mater..

[22] Koike J., Kobayashi T., Watanabe H., Suzuki M., Maruyama K., Higashi K. (2003). The activity of non-basal slip systems and dynamic recovery at room temperature in fine-grained AZ31B magnesium alloys. Acta Mater..

[23] Li N.L., Huang G.J., Xin R.L., Liu Q. (2013). Effect of initial texture on dynamic recrystallization and deformation mechanisms in AZ31 Mg alloy extruded at 573 K. Mater. Sci. Eng. A.

[24] Kim S.H., Lee S.W., Moon B.G., Kim H.S., Park S.H. (2019). Influence of extrusion temperature on dynamic deformation behaviors and mechanical properties of Mg-8Al-0.5Zn-0.2Mn-0.3Ca-0.2Y alloy. J. Mater. Res. Technol..

[25] Xu C., Zheng M.Y., Xu S.W., Wu K., Wang E.D., Fan G.H., Kamado S., Liu X.D., Wang G.J., Lv X.Y. (2013). Microstructure and mechanical properties of Mg-Gd-Y-Zn-Zr alloy sheets processed by combined processes of extrusion hot rolling and ageing. Mater. Sci. Eng. A.

[26] Jiang M.G., Xu C., Yan H., Fan G.H., Nakata T., Lao C.S. (2018). Unveiling the formation of basal texture variations based on twinning and dynamic recrystallization in AZ31 magnesium alloy during extrusion. Acta Mater..

[27] Huang K., Loge R.E. (2016). A review of dynamic recrystallization phenomena in metallic materials. Mater. Des..

[28] Hadorn J.P., Hantzsche K., Yi S., Bohlen J., Letzig D., Wollmershauser J.A., Agnew S.R. (2012). Role of solute in the texture modification during hot deformation of Mg-rare earth alloys. Metall. Mater. Trans. A.

[29] Hadorn J.P., Sasaki T.T., Nakata T., Ohkubo T., Kamado S., Hono K. (2014). Solute clustering and grain boundary segregation in extruded dilute Mg-Gd alloys. Scr. Mater..

[30] Al-Samman T., Gottstein G. (2008). Dynamic recrystallization during high temperature deformation of magnesium. Mater. Sci. Eng. A.

[31] Zhang J., Chen B., Liu C. (2014). An investigation of dynamic recrystallization behavior of ZK60-Er magnesium alloy. Mater. Sci. Eng. A.

[32] Torbati-Sarraf S.A., Sabbaghianrad S., Figueiredo R.B., Langdona T.G. (2017). Orientation imaging microscopy and microhardness in a ZK60 magnesium alloy processed by high-pressure torsion. J. Alloy. Compds.

